# Supplementary material for: Blue light-induced LOV domain dimerization enhances the affinity of Aureochrome 1a for its target DNA sequence
Source: eLife. 2016 Jan 12;5:e11860. doi: 10.7554/eLife.11860 (PMC4721966; doi:10.7554/eLife.11860)
Supplement: Figure 7—source data 1. — DOI: http://dx.doi.org/10.7554/eLife.11860.026 [file elife-11860-fig7-data1.docx]

| Sample | Concentration (mg/ml) | R_g_ (Å) (Guinier) |
| --- | --- | --- |
| *Pt*Au1a_full_ dark | 10 | 41.1 |
| *Pt*Au1a_full_ dark | 5 | 51.6 |
| *Pt*Au1a_full_ dark | 2.5 | 38.4 |
| *Pt*Au1a_full_ light | 10 | 58.1 |
| *Pt*Au1a_full_ light | 5 | - |
| *Pt*Au1a_full_ light | 2.5 | 48.4 |
| *Pt*Au1a_full_-DNA light | 5.8 | 53.2 |
| *Pt*Au1a_full_-DNA light | 2.9 | 54.9 |
