# Supplementary material for: Blue light-induced LOV domain dimerization enhances the affinity of Aureochrome 1a for its target DNA sequence
Source: eLife. 2016 Jan 12;5:e11860. doi: 10.7554/eLife.11860 (PMC4721966; doi:10.7554/eLife.11860)
Supplement: Figure 7—source data 2. — DOI: http://dx.doi.org/10.7554/eLife.11860.027 [file elife-11860-fig7-data2.docx]

| Sample | Concentration (mg/ml) | R_g_ (Å) (Guinier) | Real Space R_g_ (Å) (Gnom) | Reciprocal R_g_ (Å) (Gnom) | D_max_ (Å) (Gnom) |
| --- | --- | --- | --- | --- | --- |
| *Pt*Au1a_bZIP-LOV_ dark | 9 | 30.0 | 30.4 | 30.3 | 106.1 |
| *Pt*Au1a_bZIP-LOV_ dark | 5 | 28.1 | 27.9 | 27.9 | 97.8 |
| *Pt*Au1a_bZIP-LOV_ dark | 2.5 | 27.4 | 27.5 | 27.5 | 97.2 |
| *Pt*Au1a_bZIP-LOV_ light | 9 | 29.2 | 29.7 | 29.6 | 102.4 |
| *Pt*Au1a_bZIP-LOV_ light | 5 | 31.5 | 32.2 | 32.0 | 113.0 |
| *Pt*Au1a_bZIP-LOV_ light | 2.5 | 35.3 | 35.7 | 35.5 | 122.7 |
| *Pt*Au1a_bZIP-LOV_-DNA light | 3.1 | 38.5 | 41.1 | 40.8 | 135.1 |
| *Pt*Au1a_bZIP-LOV_-DNA light | 6.2 | 40.8 | 42.9 | 42.6 | 144.0 |
